# Psychological Factors Affecting Assertiveness in Subjects with Parkinson’s Disease

**DOI:** 10.3390/jcm13164625

**Published:** 2024-08-07

**Authors:** Viviana Lo Buono, Laura Culicetto, Matteo Berenati, Giuseppe Stroscio, Chiara Sorbera, Amelia Brigandì, Silvia Marino, Giuseppe Di Lorenzo, Angelo Quartarone, Maria Cristina De Cola

**Affiliations:** IRCCS Centro Neurolesi “Bonino-Pulejo”, S.S. 113 Via Palermo C. da Casazza, 98124 Messina, Italy; viviana.lobuono@irccsme.it (V.L.B.); matteo.berenati@irccsme.it (M.B.); giuseppe.stroscio90@gmail.com (G.S.); chiara.sorbera@irccsme.it (C.S.); amelia.brigandi@irccsme.it (A.B.); silvia.marino@irccsme.it (S.M.); giuseppe.dilorenzo@irccsme.it (G.D.L.); angelo.quartarone@irccsme.it (A.Q.); mariacristina.decola@irccsme.it (M.C.D.C.)

**Keywords:** assertiveness, anxiety, interpersonal behavior, depression, quality of life, Parkinson’s disease

## Abstract

**Background/Objectives**: Assertiveness, defined as the positive affirmation of oneself, encompasses the ability to refuse requests, express anger, disagree or oppose others, show affection, and uphold personal beliefs without causing conflict. Deficits in assertive behavior are often linked to pathological changes in the basal ganglia and prefrontal dopaminergic systems, commonly observed in Parkinson’s disease (PD), and are predictive of poor clinical outcomes. Psychological factors such as mood alterations and cognitive dysfunction may also impact assertiveness. This study investigated the psychological factors influencing assertiveness in individuals with PD. **Methods**: A cross-sectional study was conducted, involving 160 patients with PD attending a movement disorders outpatient clinic. The participants underwent assessment using a battery of standardized neuropsychological tests to evaluate cognitive function, assertiveness, mood, dysarthria, and quality of life (QoL). **Results:** All dimensions of assertiveness correlated with depression and anxiety. Individuals experiencing mood disturbances may struggle to express themselves assertively. Similarly, some dimensions of assertiveness correlated also with the QoL, indicating that, overall, well-being affects assertive behavior. Gender emerged as a significant influencer of assertiveness across all dimensions. Specifically, in subjects with PD, the male gender was associated with lower scores in assertiveness compared to women. No significant correlations were found between assertiveness and dysarthria. **Conclusions**: The findings highlight the importance of adopting a holistic approach to PD management, addressing not only motor symptoms but also psychological challenges which patients may encounter in their daily lives.

## 1. Introduction

Parkinson’s disease (PD) is a neurodegenerative disorder characterized by a progressive loss of dopamine in the nigrostriatal tract, leading to the impairment of the basal ganglia circuitry in both motor and cognitive cortical pathways [1]. Among cognitive functions, the basal ganglia play a crucial role in facilitating specific abilities, particularly those related to social cognition (SC), which encompasses various aspects of understanding and responding to social interactions [2].

SC is a behavioral construct that focuses on how people process, store, and apply information about other people and social interactions [3]. It implies the ability to identify, perceive, interpret, remember, and generate behaviors in response to the intentions, emotions, and behaviors of other human beings.

From a clinical point of view, in SC, four main components are recognized: social perception (the ability to recognize and respond to social and emotional signals conveyed by facial expressions, body posture, and tone of voice); theory of mind (the ability to understand the mental and emotional states of others); empathy (the ability to understand or feel what another person is experiencing); and social behavior (the ability to interact appropriately with tact, politeness, and without crossing borders interpersonally) [4]. SC deficits have been linked to PD but have been less investigated than general cognitive processes [5]. Most studies focused on emotion recognition, theory of mind, and decision-making domains, with limited research reporting on assertiveness.

Assertiveness represents the relational competence that allows you to recognize your feelings and emotions and express them with absolute honesty to others with mutual respect [6]. The concept of assertiveness has received attention and reformulation since its earlier definition of standing up for personal rights [7]. An assertive behavior, indeed, consists of a positive affirmation of oneself; it also includes the ability to say no, feel anger, disagree or oppose agreement and support towards the other, feel affection, and keep one’s opinions without entering into conflict with others [8]. It has been shown that good self-expression has direct effects on global well-being [9]. 

Contrarily, a lack of assertiveness is associated with several psychological problems, including stress, generalized and social anxiety, depression, and panic disorder, as well as emotional instability, strained relationships, and low self-esteem [10].

Empirical research suggests that assertiveness is negatively associated with maladaptive interpersonal schemas and emotional dysfunctions [11]. Additionally, nonassertive individuals may be inhibited from expressing themselves due to concerns about interpersonal consequences [12].

Numerous factors can affect assertiveness in subjects with PD, and they can include disease severity, cognitive impairment, and mood disorders but also life satisfaction and quality of life (QoL).

This study analyzed the psychological factors underlying assertiveness in subjects with PD to provide useful information for the development of targeted interventions aimed at improving assertive skills in this population.

## 2. Materials and Methods

This cross-sectional study included 160 subjects with PD between 51 and 79 years of age who attended the movement disorders ambulatory of the IRCCS Centro Neurolesi “Bonino-Pulejo” of Messina. A detailed description of the sample is reported in Table 1.

The inclusion criteria were the following: diagnosis of idiopathic PD according to the UK Brain Bank criteria [13], Hoehn and Yahr stages [14] (Hoehn and Yahr, 1996) 2–3, and stable pharmacological treatment (dopaminergic therapy: dopamine agonists and Levo-dopa both) in the last 6 weeks. The sample recorded a disease duration of about 8 years and Montreal Cognitive Assessment (MOCA) to assess global cognitive functioning ≤ 24. The exclusion criteria were as follows: atypical Parkinsonism, PD with dementia according to Diagnostic and Statistical Manual of Mental Disorders (DSM-V) criteria [15], and other neurological or psychiatric disorders.

All the subjects signed informed consent forms in accordance with the Declaration of Helsinki. This study was approved by the Istituto di Ricovero e Cura a Carattere Scientifico (IRCCS) Centro Neurolesi “Bonino-Pulejo” Ethical Committee (approval No. 6/2016) in June 2016.

### 2.1. Assessment

The assessment was performed in “on state” and included the following tests:

- The Scale for Interpersonal Behavior (SIB) [16] was used, which measures interpersonal behavior and includes four dimensions: (i) display of negative feelings or negative assertion (NA)—requesting a change in another person’s irritating behavior, standing up for one’s rights in a public situation; (ii) expression of and dealing with personal limitations (EPL)—admitting ignorance about a topic, recognition of one’s failure or shortcomings; iii) initiating assertiveness (IA)—introducing oneself, starting a conversation; and iv) positive assertiveness (PA)—giving and receiving praise or compliments, display feelings. In addition, the scale measures the general assertiveness score (GA) as an indication of a person’s level of assertiveness across various situations and types of assertive behavior.
- The Geriatric Depression Scale (GDS) [17] and the Hamilton Anxiety Rating Scale (HAM-A) [18] were used for the evaluation of depressive and anxiety symptoms, respectively.
- The Parkinson’s Disease Quality of Life Questionnaire (PDQ-39) [19] was used to evaluate the patients’ quality of life. The PDQ-39 includes eight discrete scales: (i) mobility (MO); (ii) activities of daily living (ADL); (iii) emotional well-being (EWB); (iv) stigma (S); (v) social support (SS); (vi) cognitions (COG); (vii) communication (COM); and (viii) bodily discomfort (BD).
- Speech–language was assessed using the Robertson dysarthria profile (RT), clinical-perceptual methods exploring all the components potentially involved in speech difficulties.

### 2.2. Statistical Analysis

Analyses were performed using an open-source R4.0.5 software package, considering *p* < 0.05 as statistically significant. The Shapiro–Wilk tests showed that all the target variables under study had distributions that were significantly non-normal. Therefore, to compare continuous variables on two independent samples (i.e., gender differences), we used the Mann–Whitney U test, and Cliff’s delta was used to compute effect sizes. Linear correlations were computed by Spearman’s coefficient. To estimate how mood, dysarthria, and QoL may affect assertiveness, we fitted a series of multiple models where the dependent variable was the assertiveness dimension, and the GDS, HAM-A, RT, and PDQ-39 subscales (i.e., M, ADL, EMO, S, SS, COG, COM, BOD) were the independent variables, adjusting for sex and age. To analyze complex relationships within the data, we ran linear and nonlinear models as Generalized Additive Models (set through the mgcv package of R), which were compared by an ANOVA table.

## 3. Results

### 3.1. Assertiveness and Dysarthria

No significant correlations between SIB and RT emerged. Similarly, RT was not a significant predictor for any dimensions of assertiveness (Table 2).

### 3.2. Assertiveness and QoL

**Figure 1:**
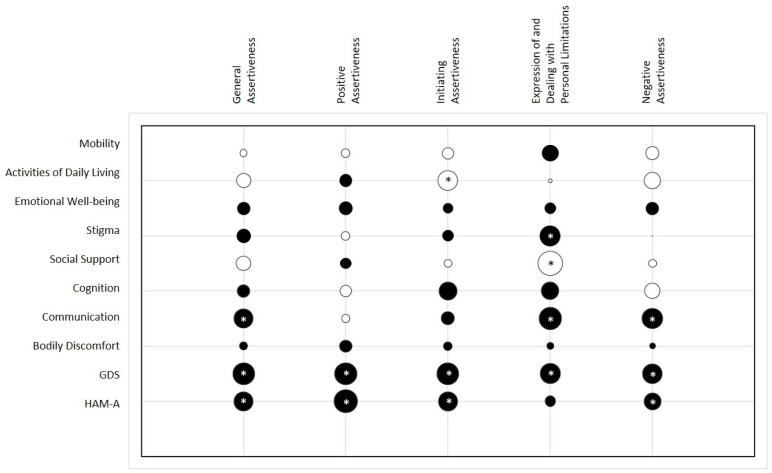
Bubble chart of correlations between assertiveness dimensions (SIB subscales), quality of life, and mood test scores. Positive correlations are in black, while negative correlations are in white. Bubble dimensions are proportional to the correlation values, and the significances are represented by asterisks centered inside the bubbles.

**Figure 2:**
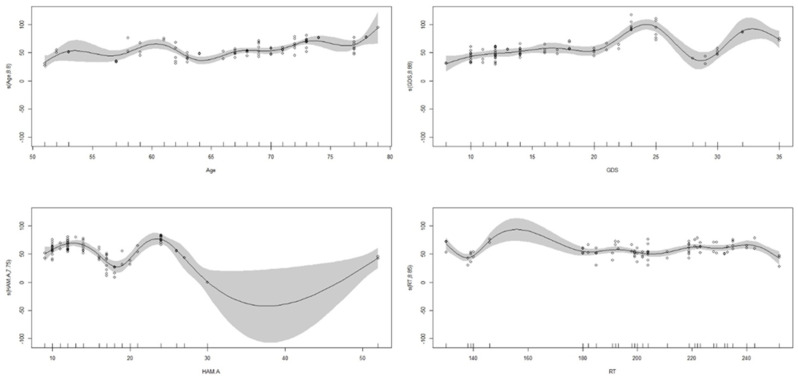
Nonlinear relationship of age, GDS, HAM-A, and PDQ-39 subscales (i.e., ADL, EMO, COG, COM, BOD) within the GAM model on general assertiveness.

We found several correlations between patients’ assertiveness and QoL, as shown in Figure 1. Notably, the highest correlations were between NA and COM (r = 0.24; *p* < 0.01), EPL and S (r = 0.26; *p* < 0.001), EPL and SS (r = −0.34; *p* < 0.001), EPL and COM (r = 0.29; *p* < 0.001), IA and ADL (r = −0.22; *p* < 0.01), and GA and COM (r = 0.20; *p* < 0.01). When we analyzed the influence of the variables on the SIB using a multivariate approach, we found that many variables had a nonlinear relationship with assertiveness, as viewable in Figure 2. Indeed, the ANOVA table suggested that incorporating nonlinear relationships between features improved the models’ explainability of the data (F = 1,507,730,296; *p* < 0.001). In particular, we found that all the PDQ-39 subscales influenced the dimensions of general assertiveness, whose scores decreased by about 0.633 ± 0.001 due to the influence of M (*p* < 0.001), by about 0.244 ± 0.001 due to the influence of S (*p* < 0.001), and by about 0.570 ± 0.001 due to the influence of SS (*p* < 0.001), as reported in Table 2.

### 3.3. Assertiveness and Mood

We found that all the subscale of assertiveness weakly correlated with the GDS—NA (r = 0.22; *p* < 0.01), EPL (r = 0.23; *p* < 0.01), IA (r = 0.27; *p* < 0.001), PA (r = 0.28; *p* < 0.001), and GA (r = 0.27; *p* < 0.001)—and a few of them with HAM-A, such as NA (r = 0.20; *p* < 0.05), IA (r = 20; *p* < 0.01), PA (r = 37; *p* < 0.001), and GA (r = 24; *p* < 0.01), as shown in Figure 1. However, as with the QoL, the relationship between depression and general assertiveness and that between anxiety and general assertiveness were not linear when considered in the GAM models (Figure 2). Notable, the GAM results showed that mood influenced all the dimensions of assertiveness, although we found that anxiety did not influence the IA (*p* = 0.179).

### 3.4. Demographics as Confounding Variables

Gender and age significantly influenced all the dimensions of assertiveness. Notably, the male gender led to a mean decrement in scores in the NA of about 10.27 ± 0.01, in the EPL of about 3.80 ± 0.02, in the IA of about 2.79 ± 0.009, and in the PA of about 3.28 ± 0.03.

## 4. Discussion

The correlations between the assertiveness dimensions and the QoL highlight the interconnectedness between these constructs. Notably, assertiveness showed significant correlations with various aspects of the QoL, including COM, SS, ADL, and GA. These findings suggest that individuals who demonstrate higher levels of assertiveness may experience better QoL outcomes, particularly in domains related to communication, social interactions, and mobility. Gender and age emerged as significant influencers of assertiveness across all dimensions. Specifically, the male gender was associated with lower scores in the NA and expressing personal limits in the EPL, IA, and PA. In other words, men with PD may be less likely to assertively express negative feelings, needs, or boundaries compared to women. This could be due to societal norms that discourage men from expressing vulnerability or discomfort openly [20]. At the same time, the decrease in scores for males in positive assertion implies that men may also struggle with assertively expressing positive feelings, appreciation, or desires. This might be influenced by societal expectations for men to suppress emotions and maintain emotional distance.

The lack of a relationship between dysarthria and assertiveness is also interesting. Dysarthria is a motor speech disorder that affects the muscles used for speech production, resulting in difficulties with articulation, pronunciation, and intelligibility [21]. It appears that, despite the communication challenges posed by dysarthria, there is not a strong association between this symptom and assertiveness levels, which involve factors such as self-confidence, social skills, and the ability to express one’s needs and opinions [8].

In line with a previous study [22], we found that anxiety and depression can significantly hinder assertiveness in people with PD.

Depression can lead to feelings of hopelessness, decreased motivation, energy, and self-confidence, which may affect assertive behavior [23,24]. People with PD might feel that they do not deserve help or that their needs are not important. Anxiety can also impair assertiveness by increasing feelings of self-doubt, fear of rejection or conflict, and avoidance of social interactions. Individuals with PD who experience anxiety may be more likely to withdraw from social situations, avoid asserting their needs, and struggle to advocate for themselves effectively [25,26].

It is possible that, in the progression of this disease, subjects with PD might tend to move from the public into the private world to mask these symptoms, with a significant and progressive reduction in their social engagement also due to stigmatization [27]. The perceived helplessness in the management of social situations could lead to or further exacerbate depressive and anxious symptoms.

Since assertiveness has traditionally been seen as the best means of expressing oneself and sustaining or increasing social benefits, it makes sense to support it as an intra and interpersonal protective factor [10]. Practicing assertiveness results in more positive interpersonal reactions [28]. In comparison to passivity or aggressiveness, assertiveness encompasses a balance between achieving personal and social goals and is, therefore, most advantageous. Since it is a respectable and culturally appropriate form of self-expression and self-affirmation [29], it enables the person to be a member of a social group while being authentically themselves. The effectiveness of human interpersonal relationships, indeed, depends on assertive reactions, which help foster greater understanding or concern from others, reciprocal respect, the maintenance of ongoing cooperative relationships with others, and, consequently, a good QoL [30]. Theatre therapy is an effective intervention for enhancing assertiveness by offering a structured, safe environment where people can explore emotions, practice social interactions, and improve communication skills. Through creative expression, role-playing, and simulations of real-life scenarios, theater therapy promotes social and emotional development, making it particularly beneficial for assertiveness training. This approach helps individuals regulate emotions, strengthen relationships, and internalize new assertive behaviors [31].

From a neurological perspective, the core of alterations in assertive behavior seems to be located in the components of the “social brain” that include the medial prefrontal cortex, the orbitofrontal cortex, the anterior cingulate cortex, the temporoparietal junction, the inferior frontal gyrus, and the superior temporal sulcus [32]. Alterations in this cerebral network have been observed in subjects with PD [33]; however, few researchers have attempted to provide a comprehensive model of assertiveness that can support the clinical importance of this construct. The ability to function successfully in social situations is highly complex and also depends on different mental abilities, such as the ability to infer the emotions of others, determine their perspectives and intentions, and appropriately use this knowledge to guide behavior and interpersonal communication [34]. Over one’s lifespan, human health and well-being also hinge on the ability to communicate effectively with others and maintain good social relationships [35]. Active screening for the non-motor spectrum, including assertive behavior, and subsequent treatment should be an integral part of the clinical management of subjects with PD to improve their well-being. Adequate knowledge of the constructs involved in assertiveness could have important implications, both clinical and rehabilitative [36].

This study has several potential limitations that may affect the validity and generalizability of its findings. One of the main limitations is the lack of assessment of the socioeconomic level of the sample, an important factor which is known to influence the course of some diseases by acting on various environmental and social factors [37]. Research on socioeconomic status in relation to disease risk is important in terms of formulating new hypotheses about the role of the environment and social factors in disease etiology [38,39,40]. Socioeconomic disparities among subjects with PD may be because of problems in diagnosis, access to care, physician referrals, and patient attitudes regarding the appropriate threshold for seeking treatment at a specialized center [41]. Another important limitation concerns the potential impact of cultural variations. Our sample predominantly consisted of individuals from a single cultural background, so the results might not accurately reflect the experiences of people with PD belonging to different cultures. Since cultural aspects can significantly influence social behavior and assertiveness, it is crucial to consider these factors in future research to ensure a comprehensive understanding [42,43]. Moreover, the impact of different pharmacological treatments and comorbid conditions on assertiveness in patients with PD has not been investigated. Another limitation is that the severity of disease progression was not considered in this study. Emotional variables may vary significantly across different stages of Parkinson’s disease [44,45,46], potentially impacting assertiveness and the overall QoL. As the disease progresses, patients may experience a decline in cognitive and emotional functioning, which could alter their ability to engage in assertive behaviors.

Concurrent medical disorders were not adequately considered. Comorbid conditions, such as cardiovascular diseases, diabetes, and other chronic illnesses [47,48,49], can significantly contribute to additional physical and psychological stress, exacerbating Parkinson’s symptoms and complicating assertive behavior.

In addition, although the sample size is sufficient, there is no indication of how well the sample represents the larger community of people with PD.

Future studies should include the analyses of these variables since they have the potential to introduce bias into the results and, as such, should be accounted for. Despite these limitations, this study has several strengths. It is one of the few studies to thoroughly investigate assertiveness in patients with PD, an aspect often overlooked in the literature. Further, the use of a variety of standardized and validated assessment tools adds robustness to the findings.

The clinical implications of this study are significant for the treatment and management of patients with PD. Enhancing assertiveness skills may positively impact patients’ QoL by improving social interactions and reducing symptoms of depression and anxiety. Targeted psychoeducation for family members could be crucial in supporting patients, enhancing social support, and reducing the stigma associated with PD [50,51]. Therapists should focus on the emotional aspects of PD, integrating assertiveness training into rehabilitation programs to help patients express their needs and emotions more effectively.

## Figures and Tables

**Table 1:** Demographic and clinical description of the sample.

Characteristics	All (*n* = 160)	Males (*n* = 88)	Females (*n* = 72)	Mann–Whitney U *p*-Value	Effect Size
Age (years)	67.47 ± 6.99	68.55 ± 4.91	66.17 ± 8.76	0.18	0.12 (negligible)
Education (years)	8.42 ± 3.73	9.00 ± 4.03	7.71 ± 3.20	0.08	0.15 (small)
MOCA score	26.00 ± 2.48	26.40 ± 2.17	25.52 ± 2.75	0.03	0.19 (small)
GA score	57.06 ± 18.93	53.43 ± 16.17	61.50 ± 21.12	0.03	0.20 (small)

Continuous variables are expressed as mean ± standard deviation, whereas categorical variables as frequencies and percentages.

**Table 2:** Generalized Additive Model results, including mood, dysarthria, and QoL, as predictors for general assertiveness, adjusted for sex and age. F-values, t-values, and *p*-values are statistics provided by GAM analyses to measure how the parameters fit the model.

General Assertiveness		Estimate	Std. Err.	t-Value	*p*-Value
Parametric coefficients	SEX	−0.192	0.031	−640.119	<0.001 ***
	RT	<0.001	<0.001	0.404	0.687
	M	−0.633	<0.001	−657.446	<0.001 ***
	S	−0.244	<0.001	437	<0.001 ***
	SS	−0.570	<0.001	−708.703	<0.001 ***
		**edf**	**Ref.df**	**F-value**	***p*-value**
Approximate significance	AGE	8.35	8.988	822,111	<0.001 ***
of smooth terms	GDS	6.334	7.176	134,752	<0.001 ***
	HAM.A	5.478	6.092	31,187	<0.001 ***
	ADL	8.843	6.056	116,810	<0.001 ***
	EMO	2.736	1.892	527,280	<0.001 ***
	COG	1.927	5.206	117,226	<0.001 ***
	COM	4.178	3.252	37,874	<0.001 ***
	BOD	2.104	2.285	358,791	<0.001 ***
**Negative Assertiveness**		**Estimate**	**Std. Err.**	**t-value**	***p*-value**
Parametric coefficients	SEX	−10.269	0.012	−1088.295	<0.001 ***
	RT	<−0.001	<0.001	−0.774	0.441
	M	−0.411	<0.001	−1600.984	<0.001 ***
	S	−0.0156	<0.001	−57.198	<0.001 ***
	SS	−0.124	<0.001	−468.754	<0.001 ***
	COG	−0.026	<0.001	−70.744	<0.001 ***
		**edf**	**Ref.df**	**F-value**	***p*-value**
Approximate significance	AGE	4.993	5.031	1,017,983	<0.001 ***
of smooth terms	GDS	8.723	8.725	983,347	<0.001 ***
	HAM.A	5.281	5.425	112,053	<0.001 ***
	ADL	7.259	7.273	1,124,527	<0.001 ***
	EMO	2.934	2.953	228,655	<0.001 ***
	COM	8.32	8.338	865,088	<0.001 ***
	BOD	1.436	1.439	5,056,902	<0.001 ***
**Expression of and Dealing with Personal Limitations**		**Estimate**	**Std. Err.**	**t-value**	***p*-value**
Parametric coefficients	SEX	−3.800	0.022	−175.527	<0.001 ***
	RT	<0.001	<0.001	0.228	0.82
	M	−0.118	0.001	−112.381	<0.001 ***
	S	0.102	<0.001	185.877	<0.001 ***
	COM	0.044	<0.001	59.821	<0.001 ***
		**edf**	**Ref.df**	**F-value**	***p*-value**
Approximate significance	AGE	5.055	5.06	147,486	<0.001 ***
of smooth terms	GDS	8.96	8.96	55,360	<0.001 ***
	HAM.A	3.945	4.417	10,086	<0.001 ***
	ADL	8.997	8.997	73,463	<0.001 ***
	EMO	1.248	1.249	149,054	<0.001 ***
	SS	3.268	3.271	115,617	<0.001 ***
	COG	5.224	5.228	33,082	<0.001 ***
	BOD	2.343	2.346	74,014	<0.001 ***
**Initiating Assertiveness**		**Estimate**	**Std. Err.**	**t-value**	***p*-value**
Parametric coefficients	SEX	−2.797	0.009	−282.776	<0.001 ***
	HAM-A	<0.001	<0.001	1.353	0.179
	RT	<−0.001	<0.001	−0.002	0.999
	M	<−0.001	<0.001	−318.502	<0.001 ***
	EMO	0.147	<0.001	508.675	<0.001 ***
	S	0.0195	<0.001	82.947	<0.001 ***
	SS	−0.146	<0.001	−524.346	<0.001 ***
	COM	0.036	<0.001	93.559	<0.001 ***
		**edf**	**Ref.df**	**F-value**	***p*-value**
Approximate significance	AGE	4.87	4.874	52115	<0.001 ***
of smooth terms	GDL	9	9	1,021,956	<0.001 ***
	ADL	9	9	1,182,855	<0.001 ***
	COG	6.435	6.44	201,908	<0.001 ***
	BOD	4.707	4.71	55,159	<0.001 ***
**Positive Assertiveness**		**Estimate**	**Std. Err.**	**t-value**	***p*-value**
Parametric coefficients	SEX	−3.280	0.036	−522.644	<0.001 ***
	AGE	0.407	0.006	911.273	<0.001 ***
	RT	<0.001	<0.001	−0.363	0.717
	ADL	0.198	<0.001	1112.535	<0.001 ***
	EMO	0.006	<0.001	39.532	<0.001 ***
		**edf**	**Ref.df**	**F-value**	***p*-value**
Approximate significance	GDS	9	9	1,285,156	<0.001 ***
of smooth terms	HAM-A	5.577	5.646	411,908	<0.001 ***
	M	1.828	1.828	993,092	<0.001 ***
	S	1.607	1.611	79,949	<0.001 ***
	SS	7.726	7.728	1,691,584	<0.001 ***
	COG	3.295	3.297	668,192	<0.001 ***
	COM	3.992	4.016	619,929	<0.001 ***
	BOD	6.933	6.937	266,505	<0.001 ***

LEGEND: GDS = Geriatric Depression Scale; HAM-A = Hamilton Anxiety Rating Scale; RT = Robertson dysarthria profile; and M = mobility. Significance codes: *** *p* < 0.001.

## Data Availability

The data supporting the findings of this study are available upon request from the corresponding author. The data are not publicly available due to privacy or ethical restrictions.
